# Supplementary material for: A high-resolution mRNA expression time course of embryonic development in zebrafish
Source: eLife. 2017 Nov 16;6:e30860. doi: 10.7554/eLife.30860 (PMC5690287; doi:10.7554/eLife.30860)
Supplement: Supplementary file 6. [file elife-30860-supp6.zip › biolayout-clusters-files/Cluster226-genes.html]

Cluster226


# Cluster226: Genes

| Ensembl ID | Gene Name | Chr | Start | End | Biotype |
| --- | --- | --- | --- | --- | --- |
| ENSDARG00000105491 | arhgef5 | 16 | 17707386 | 17730487 | protein\_coding |
| ENSDARG00000035400 | btf3 | 5 | 34387295 | 34397477 | protein\_coding |
| ENSDARG00000103670 | cmc1 | 16 | 55090608 | 55108371 | protein\_coding |
| ENSDARG00000077533 | eif3f | 1 | 9612916 | 9619933 | protein\_coding |
| ENSDARG00000004840 | rassf1 | 22 | 10615406 | 10628838 | protein\_coding |
| ENSDARG00000059298 | spcs1 | 11 | 3970016 | 3974351 | protein\_coding |
